# Improved Method of Amputation of the Penis

**Published:** 1889-11

**Authors:** 


IMPROVED METHOD OF AMPUTATION OF THE PENIS.—The *Hospital Gazette* gives the following:

The most unpleasant symptom following amputation of the penis is the funnel-like retraction of the urethra, which causes the patient endless discomfort. Dr. Assaky overcomes this by dissecting the pars spongiosa of the urethra from the penis, for about an inch beyond the superficial incision. Then the corpora cavernosa of the penis are cut through from below, leaving about an inch of the urethra protruding beyond. The tunica albuginea and the skin are then brought over the stump of the pars cavernosa and stitched around the protruding urethra. This will retract to some extent, leading a very natural looking meatus.

